# The Receptor for Advanced Glycation Endproducts (RAGE) and Its Ligands S100A8/A9 and High Mobility Group Box Protein 1 (HMGB1) Are Key Regulators of Myeloid-Derived Suppressor Cells

**DOI:** 10.3390/cancers15041026

**Published:** 2023-02-06

**Authors:** Suzanne Ostrand-Rosenberg, Tom Huecksteadt, Karl Sanders

**Affiliations:** 1 Department of Pathology, Huntsman Cancer Institute, University of Utah, Salt Lake City, UT 84112, USA; 2 George E. Wahlen Veterans Affairs Medical Center, Salt Lake City, UT 84148, USA; 3 Division of Respiratory, Critical Care, and Occupational Pulmonary Medicine, Department of Internal Medicine, University of Utah, Salt Lake City, UT 84132, USA

**Keywords:** tumor-induced immune suppression, alarmins, damage-associated molecular patterns, tumor progression, antitumor immunity

## Abstract

**Simple Summary:**

Cancer immunotherapies using antibodies and genetically modified T cells are effective in a subset of cancer patients. However, many cancer patients do not respond to immunotherapy because their cancers induce cells called myeloid-derived suppressor cells (MDSCs) that antagonize the immune system. Clinical and animal studies indicate that non-responding cancer patients have elevated levels of MDSCs. Therefore, a better understanding of the mechanisms that drive the accumulation and function of MDSCs may lead to improved cancer therapies. This article summarizes the role of an important receptor (RAGE: receptor for advanced glycation endproducts) and its two dominant ligands (S100A8/A9 and high mobility group box protein 1 (HMGB1)) that induce the accumulation and increase the immune suppressive function of MDSCs. S100A8/A9 and HMGB1 are potential biomarkers for the accumulation of MDSCs and their neutralization and/or the inhibition of RAGE may enhance cancer immunotherapies.

**Abstract:**

Immunotherapies including checkpoint blockade immunotherapy (CBI) and chimeric antigen receptor T cells (CAR-T) have revolutionized cancer treatment for patients with certain cancers. However, these treatments are not effective for all cancers, and even for those cancers that do respond, not all patients benefit. Most cancer patients have elevated levels of myeloid-derived suppressor cells (MDSCs) that are potent inhibitors of antitumor immunity, and clinical and animal studies have demonstrated that neutralization of MDSCs may restore immune reactivity and enhance CBI and CAR-T immunotherapies. MDSCs are homeostatically regulated in that elimination of mature circulating and intratumoral MDSCs results in increased production of MDSCs from bone marrow progenitor cells. Therefore, targeting MDSC development may provide therapeutic benefit. The pro-inflammatory molecules S100A8/A9 and high mobility group box protein 1 (HMGB1) and their receptor RAGE are strongly associated with the initiation and progression of most cancers. This article summarizes the literature demonstrating that these molecules are integrally involved in the early development, accumulation, and suppressive activity of MDSCs, and postulates that S100A8/A9 and HMGB1 serve as early biomarkers of disease and in conjunction with RAGE are potential targets for reducing MDSC levels and enhancing CBI and CAR-T immunotherapies.

## 1. Introduction

Checkpoint blockade immunotherapy (CBI) and chimeric antibody receptor T cells (CAR-T) have had an enormous impact on the survival of patients with a variety of solid and hematopoietic cancers. However, there remain a significant portion of cancer patients who are non-responsive to these immunotherapies. Mouse tumor studies have demonstrated that CBI and CAR-T therapy are significantly more effective when combined with approaches to reduce the levels of myeloid-derived suppressor cells (MDSCs) [1,2,3,4,5]. MDSCs are potent immune suppressive cells that profoundly inhibit antitumor immunity and promote tumor progression through a variety of mechanisms that include blocking T cell and NK cell activation and function, inducing T regulatory cells, promoting pro-tumor macrophage activity, and facilitating angiogenesis and increasing cancer cell stemness [6]. Importantly, clinical studies have confirmed that MDSCs are a significant obstacle to efficacious CBI. Studies in melanoma and prostate cancer patients documented a strong positive correlation between low levels of MDSCs and efficacy of the CBI antibody ipilimumab (anti-CTLA4) [7,8] and a lack of responsiveness to ipilimumab and the CBI antibody nivolumab (anti-PD-1) in melanoma patients with high levels of MDSCs [9,10,11]. Low levels of MDSCs in melanoma and non-small-cell lung cancer (NSCLC) patients at the start of therapy and during the early course of therapy are predictive of better responses to CBI antibodies [12,13,14]. A recent clinical trial in stage IV melanoma patients combining all-trans retinoic acid (ATRA), an inhibitor of MDSCs [15], with a CBI anti-PD-1 antibody (pembrolizumab) reduced circulating MDSC levels and resulted in a one-year overall survival rate of 80% [16]. Collectively, these experimental and human studies indicate that MDSCs are a major deterrent to efficacious cancer immunotherapies and that the elimination and/or reduction of MDSCs in cancer patients is likely to significantly enhance the efficacy of CBI.

Strategies for eliminating MDSCs include drugs that (i) differentiate MDSCs into non-immune-suppressive cells (e.g., ATRA [17]); (ii) kill MDSCs (e.g., gemcitabine and 5-fluorouracil [18,19]); (iii) inactivate MDSC-suppressive molecules (e.g., NOX2 and arginase 1 (Arg1) inhibitors [20,21]); (iv) impair signaling mechanisms in MDSCs (e.g., STAT3 [22]); as well as peptibodies [23] and monoclonal antibodies targeting myeloid cells [24], among others. However, the depletion of established MDSCs homeostatically feeds back to the bone marrow to increase MDSC development [25,26]. Therefore, short-term therapy with the above strategies may not prevent the resurgence and subsequent accumulation of MDSCs, and strategies that target the initial development of MDSCs may be more effective.

**Figure 1:**
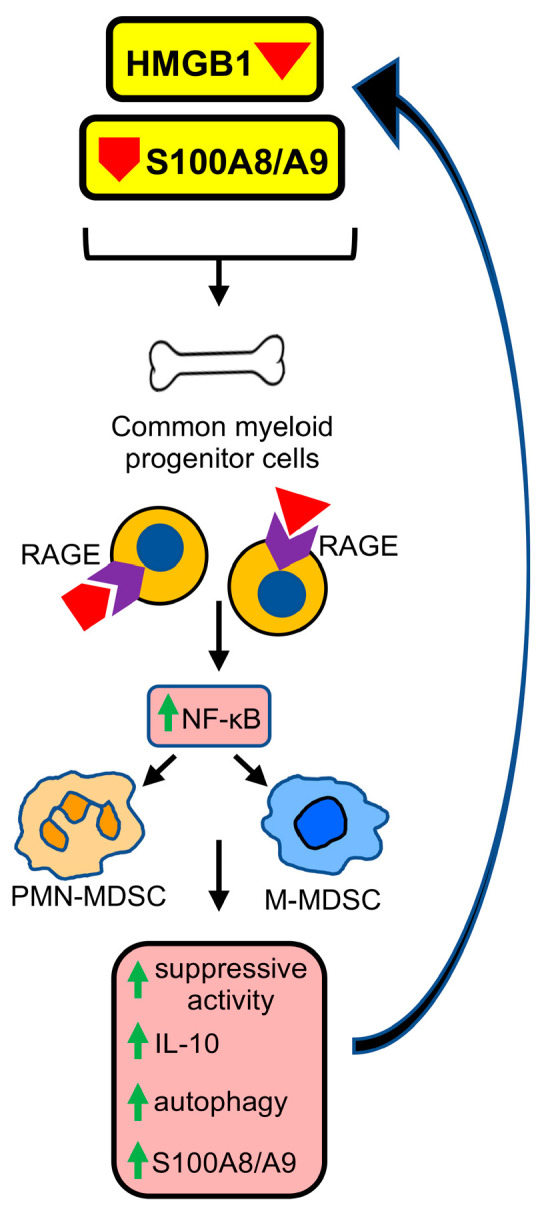
HMGB1 and S100A8/A9 induce the development of MDSCs from bone marrow progenitor cells. In healthy, non-stressed conditions, common myeloid progenitor cells (CMPs) in the bone marrow differentiate into dendritic cells, granulocytes (basophils, eosinophils, neutrophils), and macrophages. The tumor microenvironment (TME) or other stressful conditions induce damage associated molecular patterns (DAMPs) and alarmins HMGB1 and/or S100A8/A9, which bind to RAGE, and CMP differentiation is skewed toward the development of MDSCs. HMGB1 and S100A8/A9 binding activates NF-kB, resulting in increased suppressive potency, higher production of IL-10, a more autophagic state, and upregulation of S100A8/A9 by the MDSCs. The S100A8/A9 generated by MDSCs promotes the differentiation of additional CMPs into monocytic and polymorphonuclear MDSCs (M-MDSCs and PMN-MDSCs, respectively) via a feed-forward mechanism.

The receptor for advanced glycation endproducts (RAGE) and its two dominant ligands S100A8/A9 and high mobility group box protein 1 (HMGB1) have been implicated as regulatory elements promoting tumor progression in hundreds of studies in both human and mouse systems. These molecules are also associated with the initial development of MDSCs from bone marrow progenitor cells, and they regulate the suppressive potency of MDSCs (Figure 1). Therefore, RAGE, S100A8/A9, and HMGB1 may be potential targets for limiting MDSC-mediated immune suppression and promoting antitumor immunity. This article reviews the involvement of RAGE, S100A8/A9, and HMGB1 in tumor progression and how these molecules impact MDSC development, half-life, and function.

## 2. S100A8 and A9 Are Pro-inflammatory Proteins

**Figure 2:**
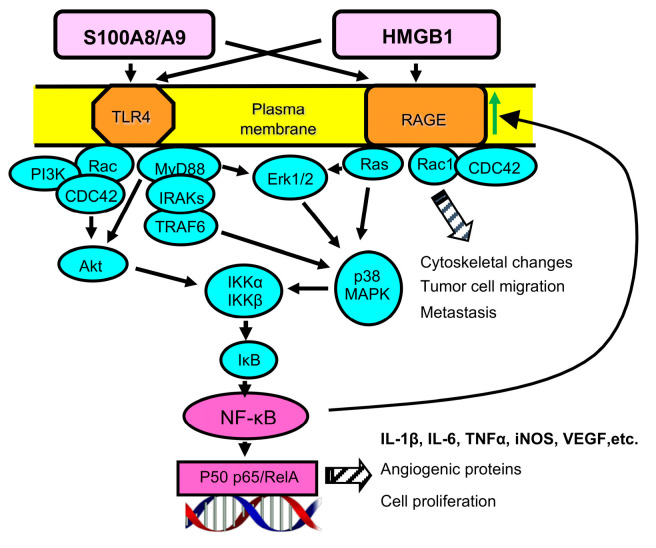
HMGB1 and S100A8/A9 are the dominant ligands for the plasma membrane receptors TLR4 and RAGE. When HMGB1 and S100A8/A9 bind to either TLR4 or RAGE, a complex intracellular signaling cascade is initiated, culminating in the translocation of NF-kB to the nucleus, where it initiates the synthesis of pro-inflammatory mediators and pro-angiogenic proteins and drives cell proliferation. NF-kB activation initiates a feed-forward loop, increasing RAGE expression (indicated by a green arrow). S100A8/A9 and HMGB1 may also individually induce crosstalk between TLR4 and RAGE, thereby simultaneously activating both signal transduction pathways.

S100A8 and A9 (also called myeloid-related proteins 8 and 14 or calprotectin A and B) are intracellular calcium binding proteins that are released extracellularly in response to several stimuli including pro-inflammatory mediators such as tumor necrosis factor (TNF) and are present at high levels in inflammatory settings. They exist predominantly as a heterodimer of S100A8/A9 but also form homodimers and tetramers [27,28]. The proteins are expressed in myeloid cells including neutrophils, other granulocytes, and immature macrophages, as well as in other cells including tumor cells [29]. S100A8 and A9 play an essential role in promoting and sustaining inflammation in the many disease settings in which inflammation occurs [30,31]. The extracellular heterodimer binds to cell membrane TLR4 [32] or RAGE [33,34] and triggers the respective signal transduction pathway, resulting in the production of reactive oxygen species (ROS); nitric oxide (NO); and the pro-inflammatory cytokines IL-1β, IL-6, and TNFα. The TLR4 and RAGE signal transduction pathways converge upon kinase signaling steps, which ultimately result in NF-κB nuclear translocation and expression of target genes [35] (Figure 2). S100A8/A9 has also been reported to stimulate crosstalk between TLR4 and RAGE, such that the signal transduction pathways may simultaneously be activated by a single ligand [36].

S100A9, but not the S100A8/A9 heterodimer, has also been reported to bind to the Emmprin/Basigin (CD147) receptor [37]. S100A8 and A9 also mediate leukocyte migration, chelate zinc and manganese to reduce microbial growth, and inhibit matrix modifying enzymes including matrix metalloproteases such as MMP9. Under normal physiological conditions, S100A8 and A9 are present at very low levels in the serum. However, their levels are significantly increased in individuals with diseases associated with inflammation, and the magnitude of the increase is a biomarker for disease severity [31,38].

In addition to their extracellular activity as pro-inflammatory mediators, S100A8 and A9 mediate effects intracellularly, where they modulate the cytoskeleton and impact microtubule polymerization and neutrophil chemotaxis and migration [39]. In vitro studies indicate that S100A8 and A9 also have anti-inflammatory activity and can downregulate pro-inflammatory cytokines, chemokines, ROS, and NO, as well as prevent mast cell degranulation [31]. This latter effect presumably protects against excessive inflammation and typically occurs during the late stages of infection/inflammation.

## 3. S100A8 and A9 Are Widely Expressed in Cancer and Strongly Associated with Tumor Progression

S100A8/A9 are overexpressed in the tumor microenvironment (TME) of many solid tumors and are present in tumor cells, infiltrating host cells including leukocytes, and plasma of cancer patients, where their expression level positively correlates with tumor progression and metastasis. For example, the heterodimer is upregulated in prostatic intraepithelial neoplasia, particularly in high-grade adenocarcinomas, and serum levels of S100A9 are significantly elevated in prostate cancer patients as compared with healthy controls [40]. Levels of S100A8/A9 are also elevated in the plasma of patients with gastric cancer [41] and glioblastoma [42] and positively correlate with stage of disease. Elevation of S100A8 in the cancer cells and stroma is associated with poor prognosis in early-stage breast cancer patients [43]. In bladder cancer patients, elevated levels of S100A8/A9 in the serum and urine correlate with bladder wall muscle invasion and reduced survival time [44,45]. Studies of patients with HPV-positive penile cancer identified S100A8/A9 in the TME and lymph node metastases, and the levels correlate with disease severity [46].

## 4. S100A8/A9 Drives the Accumulation and Potency of MDSCs in Cancer

**Figure 3:**
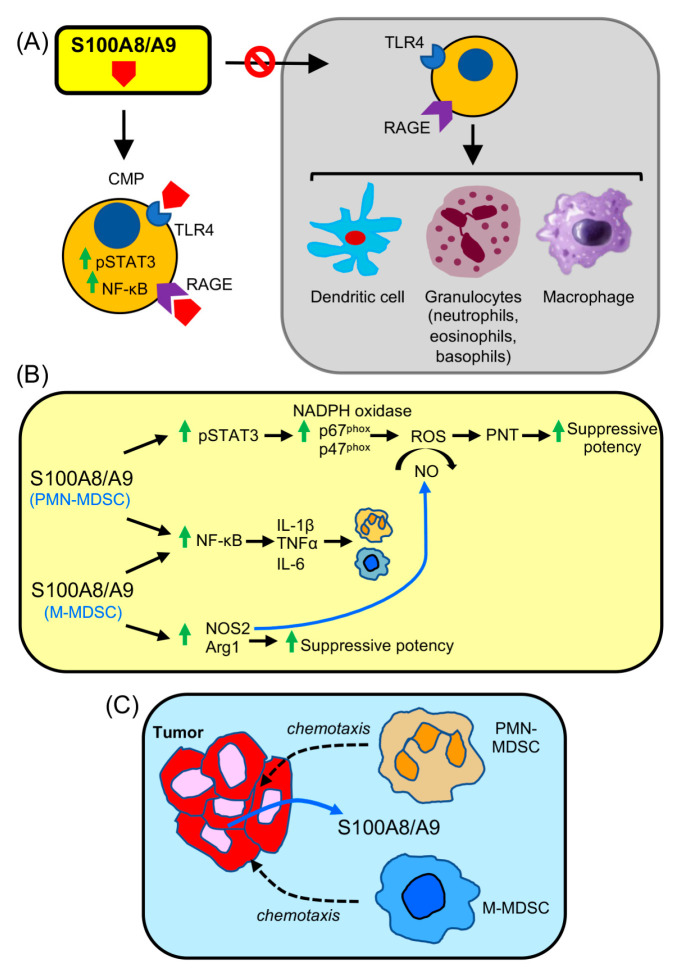
S100A8/A9 drives the differentiation, accumulation, and suppressive potency of MDSCs. (**A**) In normal myelopoiesis, CMPs differentiate into dendritic cells, macrophages, and granulocytes (neutrophils, eosinophils, and basophils). If S100A8/A9 is present, such as during inflammation or stress situations, then S100A8/A9 engages RAGE or TLR4 receptors on CMPs. The STAT3 and NF-κB transcription pathways are then activated by phosphorylation and translocation to the nucleus, respectively. (**B**) In PMN-MDSCs, activation of STAT3 upregulates the NADPH oxidase subunits p67^phox^ and p47^phox^ within PMN-MDSCs resulting in the production of reactive oxygen species (ROS). ROS enables nitric oxide (NO) to react with superoxide to produce peroxynitrite (PNT), which nitrates T cell receptors, MHC molecules, and chemokines resulting in dysfunctional T cells and blocking chemokine-mediated T cell chemoattraction. NF-κB activation results in the synthesis of IL-1β, TNFα, and IL-6, which exacerbate inflammation and further drive the accumulation of MDSCs. NF-κB is also activated in M-MDSCs along with upregulation of Arg1 and NOS2. Arg1 release depletes T cell L-arginine, an essential amino acid for T cell activation and function. NOS2 generates NO, which in conjunction with ROS produces PNT. (**C**) S100A8/A9 released by tumor cells chemoattracts MDSCs to the TME, where they suppress tumor-reactive T cells.

Multiple studies in mice and findings in patients have demonstrated that S100A8/A9 drives the accumulation, function, and migration of MDSCs (Figure 3).

Two parallel studies in mice demonstrated that S100A8 and A9 are powerful drivers for the differentiation of MDSCs from bone marrow progenitors. The induction of MDSCs involved the activation of STAT3, resulting in the synthesis of S100A8/A9 as well as activation of NF-κB within the MDSCs to enhance the MDSCs’ suppressive potency. Not surprisingly, MDSC expansion occurred at the expense of dendritic cell differentiation and function, thereby further antagonizing antitumor immunity by limiting antigen presenting cells. S100A8/A9 was also shown to be chemotactic for MDSCs and to attract MDSCs to the site of primary tumor and metastatic sites [47,48]. Mechanistically, S100A8 and A9 bind to p67^phox^ and p47^phox^, components that drive the activation of NADPH oxidase and the production of ROS [49]. Thus, S100A8 and A9 increase MDSCs’ suppressive activity by hyperactivating this pathway. Subsequent studies with a mouse model of multiple myeloma [50] and mouse thoracic tumors [51] confirmed the earlier findings that S100A8 and A9 drive MDSC differentiation and function and are associated with aggressive tumor progression.

Importantly, the mouse studies showing a key role for S100A8 and A9 have been validated for human tumors. M-MDSCs (CD33^+^CD14^+^IL-4Rα^+^ cells) are elevated in non-small-cell lung cancer (NSCLC) patients and are S100A9^+^. The M-MDSCs suppress T cells by their production of Arg1 and inducible nitric oxide synthase (iNOS or NOS2) and are correlated with overall poor prognosis and poor response to chemotherapy [52]. In an NSCLC clinical trial of 89 patients, levels of S100A9^+^ M-MDSCs (CD33^+^CD14^+^IL-4Rα^+^HLA-DR^low/−^ cells) positively correlated with metastasis and negatively correlated with response to chemotherapy and survival time. These MDSCs produced high levels of ROS [53]. In human gastric cancer, serum levels of S100A8 and A9 correlate with MDSC levels, MDSCs’ suppressive potency, and cancer stage [41]. Serum levels of S100A9 are also elevated in proportion to numbers of circulating immune suppressive PMN-MDSCs and M-MDSCs in glioblastoma patients [42]. Studies using human breast cancer cells in a xenograft model and confirmed with human clinical samples demonstrated that MDSCs produce S100A8 and are associated with poor survival and a shorter metastasis-free survival time. In the same study, a bioinformatics analysis of nine human gene expression datasets confirmed the association of increased levels of S100A8 transcripts with increased risk of death [54]. Given the direct correlation between S100A8/A9 levels, MDSC levels, and tumor progression, studies in human prostate cancer [40], colon cancer [55], and gastric cancer [41] have concluded that S100A8 and/or A9 are early biomarkers and are diagnostic for cancer progression.

Collectively, these studies demonstrate that the RAGE ligands, S100A8 and A9, produced by MDSCs and/or by tumor cells in the TME, are key molecules for driving the accumulation and function of MDSCs in individuals with cancer.

## 5. HMGB1 Is a Ubiquitously Expressed Protein with Intracellular and Extracellular Functions

HMGB1 is a non-histone chromatin-binding protein that is either resident in the nucleus or released into the extracellular space. As a nuclear protein, it binds to and stabilizes DNA and is involved in DNA repair and replication [56,57]. When HMGB1 is released from cells into the extracellular space, it serves as a damage-associated molecular pattern molecule (DAMP) and alarmin [58]. HMGB1 does not contain a secretory signal, so it is either passively released following necrotic cell death or is actively released when it translocates to cytosolic secretory lysosomes, which are subsequently exocytosed [59]. Following release and binding to one of its dominant receptors (RAGE or TLR4), downstream signaling initiates synthesis of multiple cytokines, chemokines, and other inflammation-relevant molecules [60]. As for S100A8/A9, HMGB1 may activate crosstalk between TLR4 and RAGE, thereby activating both signal transduction pathways [36] (Figure 2).

Since HMGB1 is ubiquitously expressed in the nucleus of all cells, all cells have the potential to release HMGB1. It is released from necrotic cells; however, during apoptosis, most nuclear HMGB1 remains tightly bound to DNA and, therefore, is not available for binding to plasma membrane receptors [61]. Alarmins/DAMPs such as HMGB1 are released from cells in response to danger signals and contribute to cellular repair mechanisms. However, if released in excess or if release is sustained for long periods, they contribute to chronic inflammation, which can result in tissue damage and pathogenesis [62].

**Figure 4:**
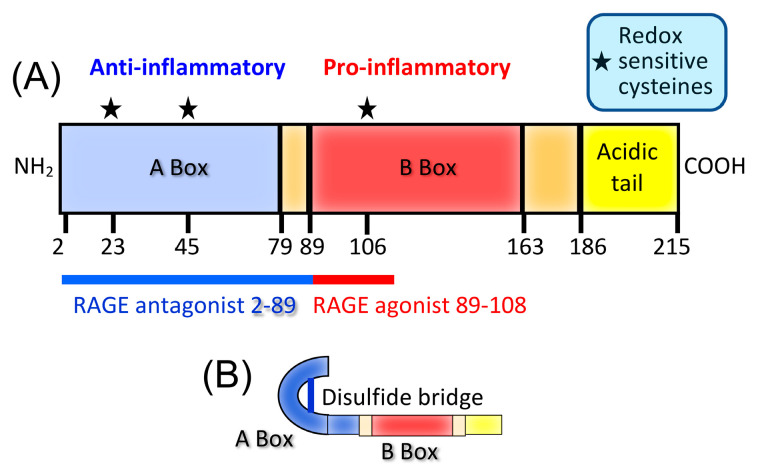
The alarmin HMGB1 contains both pro- and anti-inflammatory domains, and its action is governed by its oxidative state. (**A**) HMGB1 consists of the anti-inflammatory A box, which is a RAGE antagonist; a pro-inflammatory B box, which is a RAGE agonist; and an acidic tail. (**B**) For HMGB1 to have pro-inflammatory activity, the cysteines at positions 23 and 45 in the A box must form a disulfide bridge and the cysteine at position 106 of the B box must be fully reduced and contain a thiol side chain. In the oxidizing extracellular environment, the B box is a pro-inflammatory mediator.

HMGB1 consists of three domains: A box, B box, and C-terminal acidic domain (Figure 4A). The B box is pro-inflammatory and binds to RAGE, while the A box is anti-inflammatory and can bind to the B box. For the B box to be inflammatory and bind to its receptor, the two redox-sensitive cysteine residues in the A box must be oxidized and form an intramolecular disulfide bond (Figure 4B), presumably so the A box does not bind to and inactivate the pro-inflammatory B box. As the extracellular space is an oxidizing environment, the B box is dominant in that location, and therefore, extracellular HMGB1 is predominantly a pro-inflammatory molecule [63,64].

## 6. HMGB1 Predominantly Favors Cancer Progression

Extracellular HMGB1 is elevated in many patients and mice with cancer and is typically associated with poor prognosis and reduced survival. HMGB1 promotes human hepatocellular carcinoma invasiveness and metastases by facilitating the epithelial-to-mesenchymal transition and promoting an immune suppressive TME [65]. In vitro studies with mouse tumor cells demonstrated that this process is exacerbated within hypoxic regions of the TME because activation of hypoxia inducible factor α drives the release of HMGB1 from tumor cells [66]. The invasiveness and migration of human NSCLC cells is also mediated by HMGB1, which is released from autophagic cancer-associated fibroblasts as shown in vitro in nude mouse studies [67]. In vitro studies with an orthotopic mouse tumor, analysis of serum from breast cancer patients, and meta-analysis of human breast cancer patient data indicate that tumor-derived HMGB1 is closely associated with metastasis in triple-negative breast cancer patients [68]. Fibroblasts that promote tumor cell migration and invasiveness are also activated by HMGB1 released by a human breast cancer cell line as shown in a mouse xenograft setting [69]. HMGB1 has also been detected in tumor-cell-derived exosomes, which in combination with extracellular HMGB1 enhance cancer cell survival [70]. Indeed, HMGB1 has been implicated in the progression of a wide range of human cancers including NSCLC, pancreatic ductal adenocarcinoma, metastatic breast cancer, ovarian cancer, hepatocellular carcinoma, colorectal cancer, metastatic melanoma, mesothelioma, and gastric cancer (reviewed in [71]).

In addition to its effects on tumor cell invasiveness, HMGB1 also facilitates tumor progression by interfering with antitumor immunity. Using a three-dimensional in vitro gel system, HMGB1-treated human breast cancer cells gained enhanced migration ability and increased PD-L1 expression [72]. Studies with human colorectal cancer cells indicate that HMGB1 also reduces CD8^+^ T cell infiltration into the TME through a mechanism involving the regulation of microtubule-associated proteins [73].

Radiotherapy can provide an abscopal effect that enhances antitumor immunity by increasing dendritic cell antigen presentation [74]. However, radiotherapy causes tumor cell death and the release of HMGB1, and the extracellular HMGB1 has the potential to enhance tumor progression. For example, human pancreatic tumor cells removed from irradiated nude mice released high levels of HMGB1 when cultured. The released HMGB1 promoted cancer cell proliferation and migration in vitro. Administration of exogenous HMGB1 to tumor-bearing mice increased tumor growth [75]. Nuclear HMGB1 in radiated cells promoted DNA damage repair, thereby protecting cancer cells against some radiation-induced damage. In vivo studies with mouse bladder cancer demonstrated that inhibition of extracellular HMGB1 in combination with radiotherapy improves the radiation response [76].

Most studies are consistent with the concept that extracellular HMGB1 acting via RAGE promotes tumor progression. However, there are reports suggesting that extracellular HMGB1 reduces tumor progression. Knockdown of HMGB1 in a human lung cancer cell line resulted in increased cell migration and invasion in vitro, as well as increased metastasis in nude mice [77]. Another study showed that human NSCLC cells with reduced expression of RAGE and hence limited signaling via HMGB1 have a reduced growth rate relative to tumor cells with higher expression of RAGE [78].

HMGB1 is also a potential biomarker for cancer progression. However, whether it is a marker for high or low risk of survival is controversial. Pancreatic ductal adenocarcinoma patients with elevated serum HMGB1 levels have a poor prognosis [79]. Similarly, following several cycles of chemotherapy plus radiotherapy, lung cancer patients with high serum HMGB1 levels have shorter overall survival [80]. In contrast, high levels of plasma HMGB1 in a small group of head and neck squamous cell carcinoma patients undergoing radiochemotherapy correlated with improved survival [81]. The latter observation supports the concept that elevated HMGB1 is associated with better survival because HMGB1 is a marker of immunogenic cell death and, therefore, is associated with cytotoxic immune cell infiltrates [82,83]. Different sampling times (e.g., immediately following radiochemotherapy vs. between therapeutic interventions) could explain these apparently conflicting findings.

## 7. HMGB1 Drives the Accumulation and Function of MDSCs in Cancer

**Figure 5:**
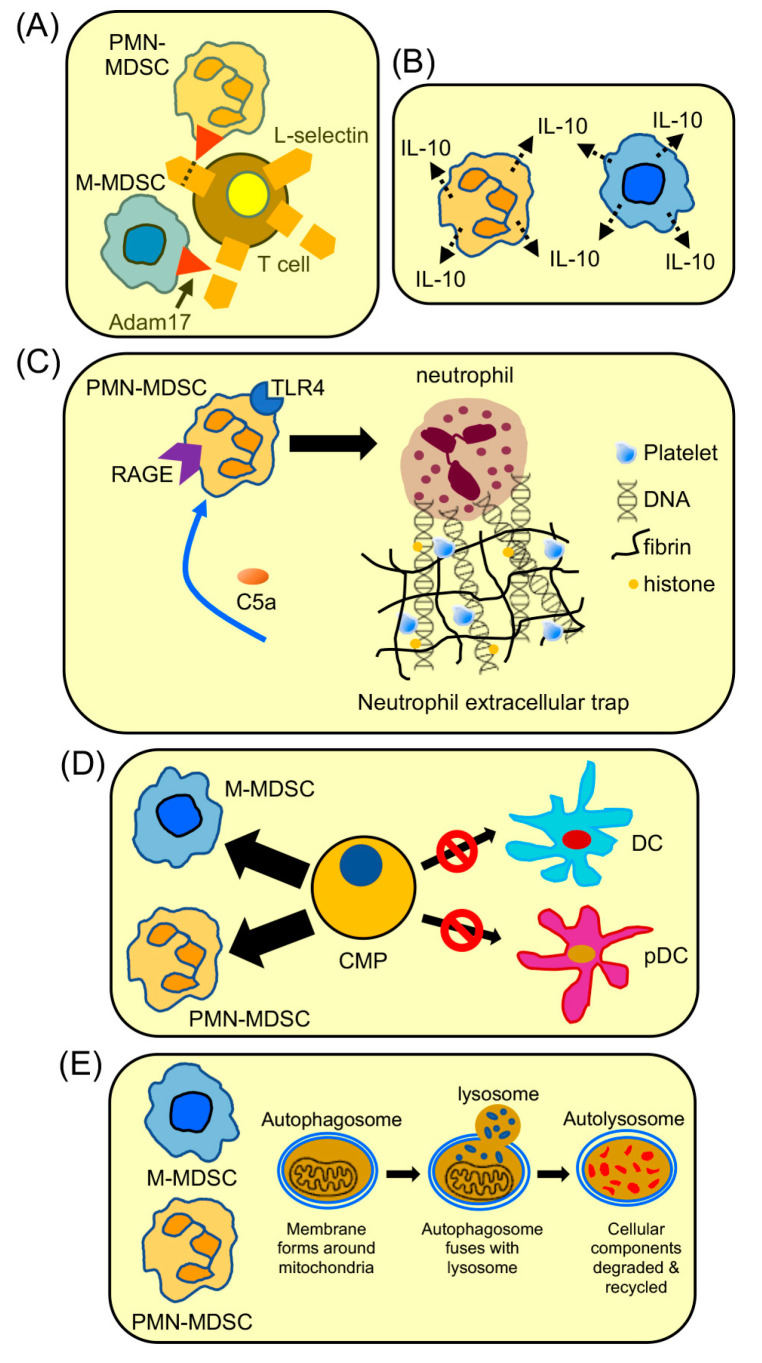
HMGB1 drives the accumulation and function of MDSCs. (**A**) Activation of T cells within lymph nodes requires that naive T cells express L-selectin so they can extravasate from the circulation and enter lymph nodes. MDSCs impair this process through their plasma membrane expression of ADAM17, an enzyme that cleaves L-selectin. L-selectin cleavage is exacerbated by HMGB1-activated MDSCs. (**B**) HMGB1 enhances MDSC production of IL-10. (**C**) HMGB1 increases PMN-MDSC expression of TLR4 and RAGE, and these MDSCs drive NETosis. MDSC-driven NETosis and MDSC migration are stimulated by complement C5a. (**D**) HMGB1 drives the differentiation of common myeloid progenitor cells (CMPs) toward MDSCs at the expense of dendritic cells (DCs) and plasmacytoid DCs (pDCs) resulting in a decrease in DCs and pDCs. (**E**) HMGB1 drives MDSC autophagy and survival.

Multiple reports demonstrate that HMGB1 is a key driver of MDSCs (Figure 5). HMGB1 is ubiquitously present in the TME of multiple mouse solid tumors [84]. Following surgical removal of colorectal tumor in a mouse system, HMGB1 levels rise dramatically and induce MDSCs. Treatment with HMGB1 A box plus gemcitabine reduces metastasis and MDSC recruitment to the peritoneal cavity [85]. Treatment of mice with renal cell tumors with antibody to HMGB1 similarly reduces tumor growth and MDSC levels. The benefit of anti-HMGB1 antibody treatment on tumor progression is diminished by prior MDSC depletion, demonstrating that MDSCs are the dominant targets for HMGB1 [86].

Key studies using multiple mouse tumors, including a triple-negative breast cancer, showed that HMGB1 drives the differentiation of MDSCs from bone marrow progenitor cells, demonstrating that HMGB1 acts at the earliest stages of MDSC development [84]. The resulting MDSCs suppress antigen-specific T cell activation, and pharmacologic inhibition of HMGB1 restores T cell activation. MDSC-mediated downregulation of L-selectin on naïve T cells, a molecule that is essential for T cells to enter lymph nodes and become activated, is also enhanced by HMGB1 [87,88]. Similarly, MDSC production of IL-10, which decreases inflammation is enhanced by HMGB1 [84].

Subsequent studies have confirmed the role of HMGB1 as a key driver of MDSC development and function and have additionally demonstrated that inhibition of HMGB1 increases antigen presentation by upregulating DCs and pDCs [89]. The latter observation supports the earlier finding that MDSCs differentiate from bone marrow progenitor cells at the expense of other myeloid cell populations [90].

Studies in a mouse lung cancer system provide evidence for another avenue by which HMGB1 drives MDSCs that contribute to metastasis. In this system, complement component C5a enhances the generation of PMN-MDSCs and increases their expression of the HMGB1 receptors TLR4 and RAGE. The resulting PMN-MDSCs induce the formation of neutrophil extracellular traps (NETs), which in conjunction with tumor-cell-released HMGB1 increase lung metastases. Inhibition of HMGB1 prevents PMN-MDSC-driven NETosis. The potential clinical relevance of this mechanism is supported by the finding that C5a stimulates the migration and NETosis by PMN-MDSCs obtained from lung cancer patients [91].

HMGB1 also plays an important role in MDSC survival. The TME is a hostile environment for cells due to high levels of ROS and hypoxia and limited availability of nutrients. MDSCs have a short half-life within the TME. However, their constant replenishment from bone marrow progenitors [25,26] coupled with entering an autophagic state sustains a level of MDSCs in the TME that directly correlates with tumor progression. Autophagic cells have a survival advantage because they degrade excessive and aberrant proteins while recycling amino acids that are essential for cellular metabolism [92]. In vitro and in vivo studies of breast-cancer-induced mouse MDSCs using inhibitors of autophagy and HMGB1 demonstrated that HMGB1 promotes MDSC survival by driving the cells into an autophagic state [93].

Collectively, these findings demonstrate that HMGB1 is a key ligand for MDSC development and survival, effects on T-cell-mediated antitumor immunity, and regulation of inflammation in the TME.

## 8. Receptor for Advanced Glycation Endproducts (RAGE) Is a Player in Malignancy

RAGE is a receptor for advanced glycation endproducts (AGEs), which includes a group of diverse ligands derived by non-enzymatic glycation. It is also a receptor for alarmins and DAMPs. RAGE is a member of the immunoglobulin superfamily and is intimately involved in the pathology associated with many inflammatory diseases. As described, RAGE ligands S100A8/A9 and HMGB1 are strongly implicated in many aspects of neoplasia. Binding to RAGE activates a complex intracellular signal transduction pathway culminating in activation of NF-κB, which in turn directs the synthesis of multiple pro-inflammatory mediators (Figure 2). NF-κB activation also upregulates RAGE expression, thereby generating a feed-forward loop and further activation [94]. RAGE activation was initially discovered as a central element in the induction of inflammation. Subsequent studies demonstrated that RAGE is a major contributor to tumor progression and metastasis [95,96,97]. Activation of RAGE within the TME results in multiple pro-tumor activities including (i) enhancing cancer cell invasion, dissemination, and metastasis; (ii) remodeling of the extracellular matrix to provide a scaffold for supporting tumor progression; (iii) driving inflammation that supports tumor progression and invasion; (iv) upregulating VEGF to support neo-angiogenesis; (v) metabolic reprogramming to favor growth in the hypoxic TME; (vi) counteracting genomic instability by supporting DNA repair; and (vii) driving and sustaining immune suppression (reviewed by [98]). The finding that RAGE-null mice have fewer tumors in colitis-associated cancer [34] and inflammation-induced skin cancer [96] further demonstrates that RAGE plays an important role in carcinogenesis and tumor progression.

## 9. RAGE Contributes to the Generation and Pro-Cancer Effects of MDSCs

The first indication that RAGE is involved in MDSC activation and function came from studies using spontaneously metastatic mouse triple-negative breast cancer cells [47]. Using an anti-RAGE antibody and an antibody detecting carboxylated N-glycans expressed by RAGE, in vivo induced MDSCs were shown to bind the RAGE ligand S100A8/A9 heterodimer. Both tumor-induced MDSCs and cells with the phenotype of MDSCs from tumor-free mice bound the antibodies. The MDSCs also expressed RAGE, HMGB1, and S100A8 and A9 transcripts and Western blots confirmed the presence of the corresponding proteins, although only tumor-induced MDSCs contained S100A8/A9 heterodimers. MDSC binding of S100A8/A9 resulted in the activation of NF-κB, and NF-κB activation was reduced in the presence of antibody to RAGE, thus indicating that RAGE serves as a receptor for MDSC activation. In vivo treatment of mice with metastatic lesions with the anti-RAGE antibody reduced the accumulation of MDSCs in the circulation, in draining lymph nodes, and in the spleen. Although blocking of RAGE reduced MDSC levels, it did not reduce the MDSCs’ suppressive potency.

Subsequent studies using RAGE-null mice transgenic for the Kras^G12D^ mutation that spontaneously develop pancreatic ductal adenocarcinoma confirmed the role of RAGE in MDSC biology [99]. MDSC accumulation in these mice was reduced relative to that in RAGE-sufficient littermates. In contrast to the breast cancer studies [47], RAGE deficiency reduced the MDSCs’ suppressive activity. The differences between the breast cancer study and this Kras^G12D^ study could be due to the different assays used to assess suppression and could also be due to misidentification of cells in the latter study as MDSCs. The breast cancer study used antigen-specific suppression, while the Kras^G12D^ study used global T cell suppression (anti-CD28/anti-CD3 antibodies). The reduced levels of MDSCs in the RAGE-deficient Kras^G12D^ mice resulted in higher levels of macrophages, consistent with the concept that, in the absence of activation through RAGE, bone marrow progenitor cells differentiate into macrophages instead of MDSCs [99]. The finding that MDSCs develop at the expense of other myeloid cells was also noted in an in vitro system in which MDSCs were differentiated from bone marrow progenitor cells [90].

Additional studies with RAGE-null mice and mice treated with the RAGE inhibitor TTP-488 further identified the important role of RAGE in the induction of MDSCs [100]. RAGE-null and TTP-488-treated wildtype mice had longer survival times, reduced metastatic tumor burden, and fewer MDSCs relative to wildtype and untreated mice following intravenous injection of two mouse tumors. The MDSCs that accumulated in the lungs and spleens of RAGE-null mice were less suppressive in antigen-specific T cell assays and contained reduced enzymatic activity for iNOS and Arg1 compared to MDSCs in wildtype mice. NF-κB was less activated in MDSCs from RAGE-null mice, and transcripts of the RAGE ligands S100A8/A9 and HMGB1, as well as the pro-inflammatory molecules TNFα and VEGFα, were expressed at lower levels relative to wildtype MDSCs. In agreement with other studies, reduced levels of MDSCs in RAGE-null mice were associated with increased numbers of macrophages, again consistent with the concept that the differentiation of MDSCs occurs at the expense of other myeloid cells.

Collectively, these findings along with the findings of HMGB1 and S100A8/A9 discussed in the previous sections identify HMGB1 and S100A8/A9 as critical ligands for activating MDSCs through RAGE.

## 10. Conclusions

The impact of RAGE and its principal ligands S100A8/A9 and HMGB1 on multiple aspects of tumor progression has been established in numerous and diverse mouse and human cancers. Likewise, the role of RAGE, S100A8/A9, and HMGB1 in regulating the development, accumulation, and function of MDSCs is well established. Most studies aimed at understanding the activation of MDSCs have focused on the role of pro-inflammatory factors, including S100A8/A9 and HMGB1, acting through their canonical receptors. What remains unknown is whether S100A8/A9 and HMGB1 act exclusively via RAGE to activate MDSCs or whether the ligands also bind to and activate MDSCs via their alternative receptor TLR4. It is also not clear whether there is a hierarchy for the activation of MDSCs in which certain receptors, such as RAGE, are used first. Alternatively, receptor usage for MDSC activation may be controlled by the dominant ligand present in the TME. HMGB1 and S100A8/A9 are ubiquitously present in most solid TMEs and auto-stimulate their own production, and activation via RAGE occurs early in the process of MDSC differentiation. Therefore, although there may be other MDSC-activating ligands and receptors in the TME, HMGB1 and S100A8/A9 are likely to be key ligands for activating and sustaining MDSC accumulation and driving MDSCs’ suppressive potency through their binding to RAGE. Given that activation through RAGE regulates the very early stages of MDSC development, RAGE may be a potential target for reducing MDSC-mediated immune suppression and avoiding the homeostatic feedback that leads to increased production of MDSCs. RAGE, HMGB1, and S100A8/A9 are critical molecules for MDSC development and function, but they also have other mechanisms by which they promote tumor growth. Therefore, targeting RAGE, HMGB1, and S100A8/A9 will not only reduce immune suppression and facilitate antitumor immunity but may also delay tumor progression by neutralizing the many other mechanisms by which these molecules promote tumor growth.
